# Rounding up the usual suspects: confirmation bias in epidemiological research

**DOI:** 10.1093/ije/dyab091

**Published:** 2021-04-30

**Authors:** R Scott Braithwaite, Kaoon (Francois) Ban, Elizabeth R Stevens, Ellen C Caniglia

**Affiliations:** Department of Population Health, NYU School of Medicine, New York, NY, USA

Investigators performing epidemiological research frequently form hypotheses based on data availability. One might ask how it could be otherwise. After all, what is the point of forming hypotheses if they can’t be tested? But when questions are identified to suit available data rather than data being identified to suit important questions, commonalities in measured and unmeasured variables extend across multiple studies and lead to a confirmation bias. Expected relationships are confirmed, and unexpected relationships remain undiscovered, even when their unveiling would have important informational value. We argue that this confirmation bias results from a structural cause, in particular misalignment of epidemiological research priorities with the social utility of research.

## Social utility of research

The social utility of research can be measured using value of information (VOI), which quantifies the utility of improvements in decision making that would be possible from the additional information produced by the study.^1^^,^^2^ If a study is (i) likely to produce information that leads to changes in decision making and (ii) these changes have large consequences for health and/or for resource investments, then that study will have large VOI. In contrast, if a study is unlikely to produce information that leads to changes in decision making and/or those changes would have small consequences for health and/or for resource investments, then that study will have small VOI. Often, studies will maximize VOI if they evaluate hypotheses with moderate pre-evaluation likelihoods of being proven or disproven in the context of relevant benefits, harms and costs. Here, ‘pre-evaluation likelihood’ is analogous to the idea of ‘pre-test likelihood’ regarding a diagnostic test, and reflects the probability or odds that a hypothesis is true before its evaluation by hypothesis testing, analogous to the probability or odds that a condition truly exists before its evaluation by diagnostic testing. If the pre-evaluation likelihood of being proven or disproven is too small, a confirmatory result will likely be interpreted as a false positive, and will not lead to a change in decision making. On the other hand, if the pre-evaluation likelihood is too large, this likelihood will already have affected decision making, and a confirmatory result will add little additional information to further alter decisions.

## Investigator’s utility of research

To some extent, an investigator’s utility reflects a cost-benefit calculus that mirrors VOI. If pre-evaluation likelihood of being proven or disproven too small, it will often be difficult to obtain peer-reviewed funding, and the cost to the investigator would be prohibitively high. If the pre-evaluation likelihood of being proven or disproven is too high, the result may be greeted with indifference, yielding little recognition or prestige.

However, the calculus of investigator’s cost-benefit deviates from societal VOI when existing data sources exist with low entry barriers for a particular investigator(s) but with high entry barriers for other investigators. Then, that investigator’s cost-benefit calculus strongly favours continuing to create hypotheses around that existing dataset because the cost of each marginal hypothesis test becomes extraordinarily low. Hypotheses with low or high pre-evaluation likelihoods can be tested with little additional effort, and are justified by the modest reward of publishing an unsurprising but previously unreported finding, or an expected confirmatory result in a new context.

## How cost/benefit from investigator’s perspective diverges from society’s VOI perspective

Availability of existing datasets may skew an investigator’s cost/benefit calculus away from societal utility. Data available for analysis exist because a previous decision was made that the incremental reward of future analyses was believed to exceed the incremental time, cost and effort of establishing data embedding those constructs as a substrate for analysis. Even if the data are being used for purposes outside what was originally intended, the original intent persists and is reflected by the choices made regarding what to include and what to exclude. Indeed, a dataset can be viewed as encoding not only data, but also scientific beliefs and institutional priorities at the time of its origination. These may not reflect social utility either currently or at their time of origination, especially if they were not created for purposes of research.

Datasets originating close together in time, or within a timespan without much change in funder priorities or scientific domain knowledge, are likely to bear the imprint of similar cost/benefit calculus by funders and investigators. Consequently, similar constructs will be measured in distinct datasets and in distinct analyses in the same datasets. For the same reason, similar constructs will be unmeasured in distinct datasets and in distinct analyses in the same datasets.

Moreover, investigators gravitate towards data sources embedding similar constructs for reasons beyond accessibility. Datasets funded by a particular organization will often have commonalities in organization, structure, and technical ‘know how’ required to use them; and therefore the incremental work involved in successive analyses with similar datasets is smaller than the incremental work involved in analyses of differently constructed datasets. This scale efficiency is evidenced by the numerous research laboratories that are constructed around a facility with a particular data or database type. Investigators, like all competitive agents, migrate towards situations in which barriers to entry reduce competition. Consequently, junior investigators may select to apprentice with senior investigators based on access to particular data sets.

If an association identified in an index analysis happens to represent causality, because those same variables or related concepts are particularly likely to be sampled in subsequent analyses, that causal relationship is likely to be reproduced and confirmed. However, if an association in an index analysis does not represent causality, that spurious relationship is also likely to be reproduced and confirmed because variables or related constructs contributing to the spurious relationship are absent not only in that index data source but also in subsequently available data sources.

## Example: epidemiological studies of life expectancy and education

**Figures 1:**
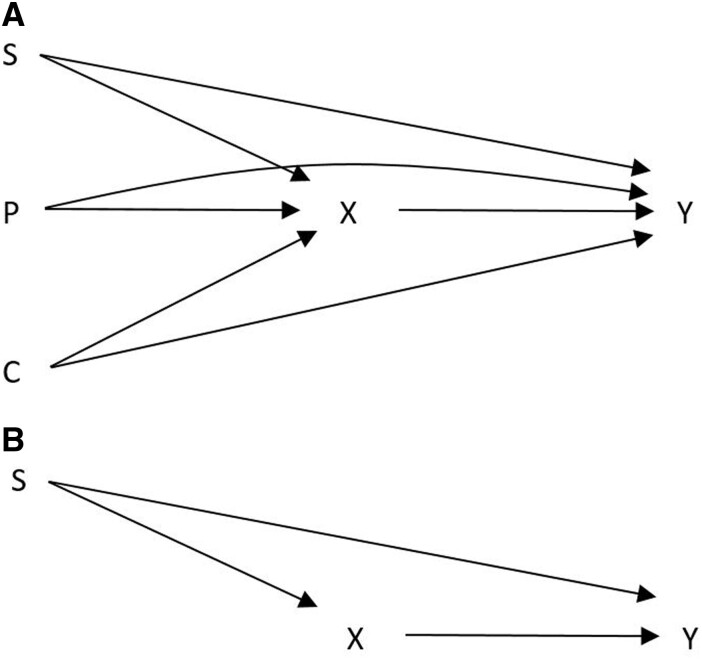
Schematic diagram depicting bias from commonalities in unmeasured constructs. Figures 1A and B depict causal diagrams for estimating the causal effect of education (X) on life expectancy (Y). S is a measured confounding construct (social support) and P (personality type) and C (inherited capital) are unmeasured confounding constructs. The true causal diagram is depicted in Figure 1A and the apparent but spurious causal diagram based on lack of information or knowledge about unmeasured constructs P and C is depicted in Figure 1B

An illustrative example may be the finding that greater education predicts longer life expectancy.^3^ According to the causal diagram depicted in Figure 1A, an estimate of the causal effect of education (*X*) on life expectancy (*Y*) will be biased due to confounding by conscientious personality type (*P*), level of social support (*S)* and quantity of inherited capital (*C*). If conscientious personality type and quantity of inherited capital are unknown and unmeasured confounders, investigators may assume Figure 1B is the correct causal diagram for estimating the causal effect of education on life expectancy, and will incorrectly conclude that adjusting for level of social support is sufficient to estimate an unbiased estimate of the causal effect of education on life expectancy.

If the associations between higher education and health are likely to represent a causal relationship, social funding of higher education would be of paramount importance for population health. However, if this relationship were substantially confounded by another variable, such as inherited wealth, the policy implication would be different. In that case, government-funded savings accounts starting at birth (‘baby bonds’) may substantially increase health but government-subsidized higher education may not. Suppose that higher education (completion of college versus no college) was associated with a gain in life expectancy of 3 years but that much of this association was not due to a causal effect of higher education on life expectancy. For example, imagine an intervention on higher education would increase life expectancy by only 0.5 years. Assume that subsidizing universal college cost $50 000 per person. On the other hand, suppose that inherited wealth ($50 000 versus $0) was associated with a gain in life expectancy of 2 years, and that an intervention on inherited wealth was entirely causal; that is, it would increase life expectancy by the full 2 years. A study adding to evidence regarding the causal relationships between education, inherited wealth and life expectancy could yield a VOI for the USA of as much as $45 trillion because additional investments considered for education (potentially adding 0.5 years) could instead be re-allocated more efficiently towards inherited wealth (potentially adding 2 years, an additional 1.5 years); and 1.5 life-years x $100 000 per added life-year x 300 million people amounts to $45 trillion). Because available datasets may not contain all relevant variables for such an analysis, the analysis may have too high of a ‘cost’ from an investigator’s perspective. However, the societal benefit could be huge. In contrast, a study adding to evidence regarding the apparent association between education and life expectancy may add to precision or generalizability of an existing estimate, for example changing the uncertainty from a previous cumulative estimate of 3 years with a previous cumulative variance of 2 years to a new cumulative estimate of 3.3 years with a new cumulative variance of 1.5 years. However, in the absence of additional inferences regarding causality, this modest increase in certainty would lead to little incremental improvement in decision making.

## Published studies on education and life expectancy

**Figure 2:**
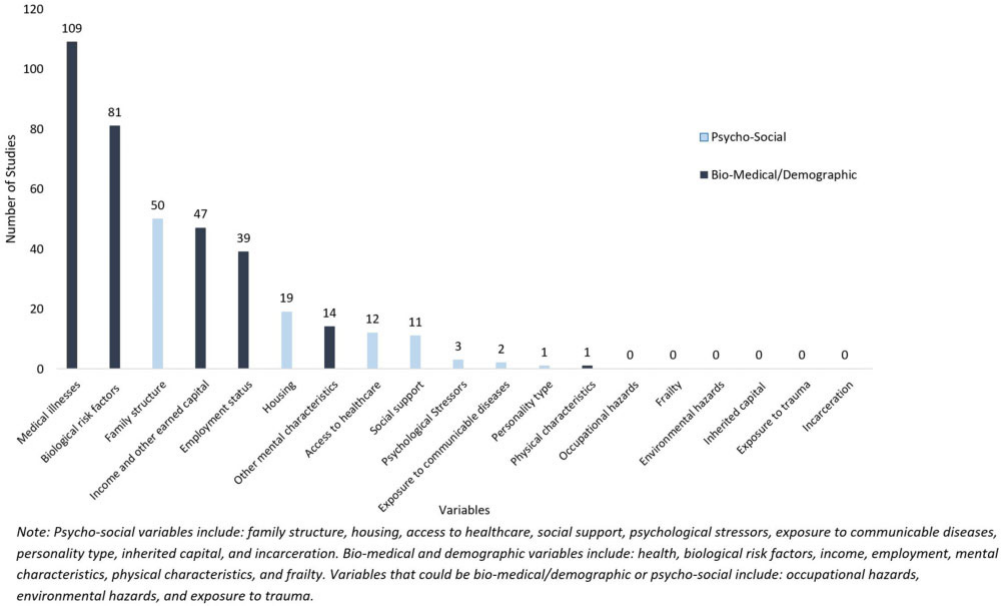
Number of studies measuring psychosocial and biomedical/demographic variables.

**Figure 3:**
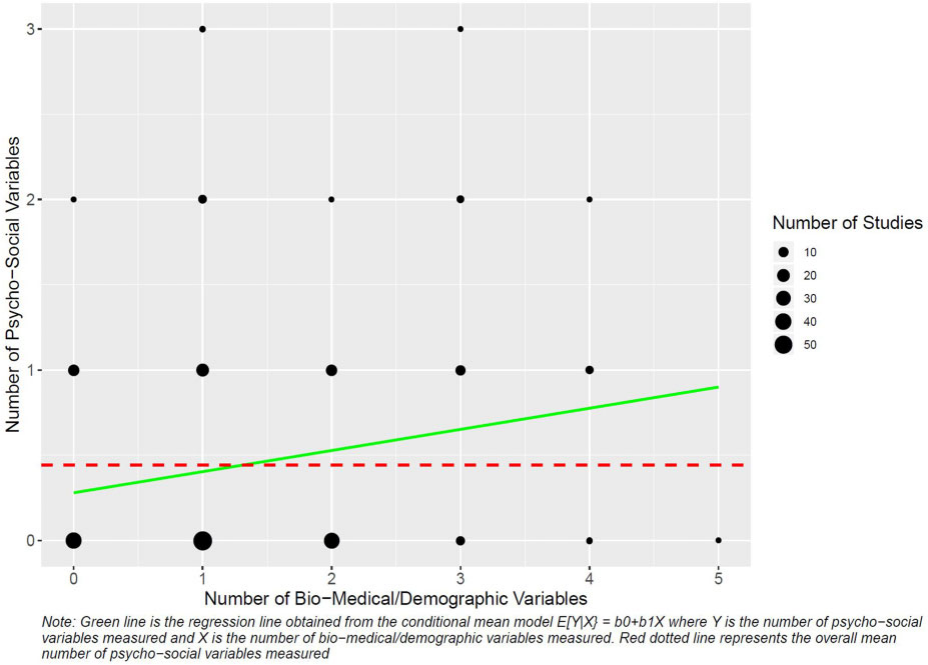
Relationship between number of biomedical/demographic variables measured and number of psychosocial variables measured across all 221 studies.

We conducted a scoping review of 221 studies reporting on the relationship between education and life expectancy (Figure 2). Variables or related concepts distinct from the fields or purposes of funders, who are typically interested in biomedical and demographic constructs, were rarely included in the studies, even in the presence of evidence that they could be confounding the relationship between education and life expectancy. Such psychosocial constructs included level of social support^4^^,^^5^ (measured in 5% of studies), conscientious personality type (measured in 0.5% of studies)^6^^,^^7^ and quantity of inherited capital (financial and social) (measured in 0% of studies), which we conceptualized as confounding the causal effect of education on life expectancy. Executive function and impulsivity were measured in none. On the other hand, biomedical and demographic constructs, such as employment (measured in 18% of studies), income (measured in 21% of studies) and health (measured in 49% of studies), were included in a much larger number of studies (Figure 2). However, constructs such as employment, income and health would typically be conceptualized as downstream effects rather than causes of education (and thus measuring and adjusting for them is not necessary to adjust for confounding). Indeed, the mean number of psychosocial constructs measured in all 221 studies was quite small (0.44), even when studies included an increasing number of biomedical or demographic constructs (Figure 3).

## How can confirmation bias be detected and mitigated?

Whereas the pitfalls of secondary data analysis are well known, we argue that their ill effects are amplified when they perpetuate analyses of a closed set of variables to the exclusion of other variables. It may also be possible to assess the risk of biased construct availability. The set of constructs known or hypothesized to potentially confound the relationship between an exposure and an outcome of interest, specified without knowledge of available datasets, can be compared with the subset of these constructs included in each available dataset. If these construct subsets are highly correlated (and do not include the complete set of confounding constructs), there is substantial concern that the overlap of constructs is driving reproducibility, regardless of whether the underlying relationship is causal or not. If the subsets are not correlated, there is less concern that the overlap of constructs is driving reproducibility, and biased construct availability is unlikely to be important.

One indicator of whether confirmation bias is occurring is asking whether a research question is formed with a particular data source in mind, or whether it would still be as scientifically meritorious when considered apart from a particular dataset. If so, it may have been specified by working backward from that data source and identifying a compatible question. The research question and related hypotheses will then be invisibly yet inextricably bound by the particular mould of measured and unmeasured constructs characterizing that data source. In contrast, if the research question was formed without a particular data source in mind, that hypothesis is less likely to be misshapen by any particular mould of measured and unmeasured constructs. Much like a distinction made between *a posteriori* and *a priori* analyses, a distinction could be made between research questions and related hypotheses formed working backwards from available data sources and their embedded constructs, and hypotheses formed working forward from scientific insights external to known data. Hypotheses formed by working backwards, whether confirmed or disproven, are more likely to be replicated using other available data sources because they will have commonalities in the domains of both measured and unmeasured constructs. On the other hand, hypotheses formed by working forwards are less likely to have results anchored on an index data source with a particular constellation of measured and unmeasured constructs.

## Summary

Biases may arise not only from which data are collected, but also from commonalities in the variables embedded in available data sources, which lead to confirmation bias. The possibility that spurious relationships are being perpetuated should be considered when a relationship is reproduced in multiple datasets with substantially overlapping variables that do not include all plausibly confounding variables. When findings are reproduced, it is important to ask if the datasets have similar constellations of measured and unmeasured variables or related concepts, because the reproducibility may be an artefact of biased availability of variables. Future work is warranted to study whether biased availability of variables underlies the phenomenon of multiple observational studies with consistent causal inferences being refuted by subsequent randomized trials.

## Conflict of interest

None declared.
